# The draft genome sequence of a carbapenem-resistant *Klebsiella pneumoniae* strain belonging to sequence type 11 and capsular type 62

**DOI:** 10.1128/mra.00981-24

**Published:** 2024-10-22

**Authors:** Linwan Zhang, Yu Feng, Yongqiang Yang, Yanling He, Zhiyong Zong

**Affiliations:** 1 Center of Infectious Diseases, West China Hospital, Sichuan University, Chengdu, China; 2 Department of Clinical Research Management, West China Hospital, Sichuan University, Chengdu, China; 3 Laboratory of Pathogen Research, West China Hospital, Sichuan University, Chengdu, China; 4 Division of Infectious Diseases, State Key Laboratory of Biotherapy, Chengdu, China; Rochester Institute of Technology, Rochester, New York, USA

**Keywords:** *Klebsiella pneumoniae*, carbapenem resistance, wound secretion

## Abstract

Carbapenem-resistant *Klebsiella pneumoniae* (CRKP) of sequence type 11 (ST11) and capsular type KL47 or KL64 are dominant in China. We report the draft genome sequence of an ST11 *bla*_KPC-2_-carrying CRKP strain belonging to uncommon capsular type KL62. This strain carries virulence factors encoding multiple iron acquisition systems and mucoid regulators.

## ANNOUNCEMENT

*Klebsiella pneumoniae* is a common Gram-negative opportunistic pathogen causing a wide range of infections, including pneumonia, bacteremia, urinary tract infections, and liver abscesses (1, 2). Carbapenem-resistant *K. pneumoniae* (CRKP) has emerged as a particular challenge due to very limited therapeutic options. In China, the predominant type of CRKP is sequence type (ST) 11 and most ST11 strains belong to capsular type KL47 or KL64 (3, 4). Here, we reported the draft genome sequence of an ST11 CRKP clinical strain, which belongs to capsular type KL62, an uncommon type within ST11 strains.

A wound secretion sample was collected from a patient with trauma-induced fractures as part of routine care for a wound infection at West China Hospital in 2022. Strain 141091 was isolated from the sample, which was initially streaked onto Luria Bertani (LB) agar and incubated at 37°C for 18 hours. The strain was identified as *K. pneumoniae* and was found to be resistant to ertapenem (MIC, ≥8 mg/L), doripenem (MIC, ≥8 mg/L), imipenem (MIC, ≥16 mg/L), and meropenem (MIC, ≥16 mg/L) by the Vitek II automated microbiology system (bioMérieux, Marcy-l’Étoile, France). The strain was subjected to genome sequencing and analyzed. This study has been approved by the Ethical Committee of West China Hospital, with informed consent being waived.

Genome DNA was extracted using the QIAamp DNA Blood Mini Kit (Qiagen, Hilden, Germany) according to the manufacturer’s instruction from an overnight culture of a single colony in LB broth. A 350 bp insert library was constructed using the NEBNext Ultra II Kit (New England Biolabs, Ipswich, MA). Sequencing on the Illumina NovaSeq 6000 platform (San Diego, CA) with 150 bp paired-end reads generated 4,566,921 read pairs (1.37 gigabytes) corresponding to an approximately 270× coverage. Raw reads underwent quality trimming with Trimmomatic v0.39 (5) and were subsequently reduced to 150× coverage depth by random subsampling with SeqKit v2.8.2 (6). Then, reads were assembled into a genome comprising 153 contigs, totaling 5,822,570 bp, with an N*50* of 176,097 bp and a GC content of 57.01%, using SPAdes v3.15.5 (7) with isolate mode enabled. The strain was precisely identified as *K. pneumoniae* (*sensu stricto*) sharing an average nucleotide identity of 98.88% with *K. pneumoniae* type strain ATCC 13883^T^ (accession no. CP064368) determined using FastANI v1.34 (8). The strain was assigned ST11 and KL62 using Kleborate v2.4.1 (9). Antimicrobial resistance genes were identified using AMRFinderPlus v3.12.8 (10). Default parameters were used for all software. The strain carries *bla*_KPC-2_, a carbapenemase gene, consistent with its carbapenem resistance phenotype. Virulence factors were predicted using Kleborate v2.4.1 (9). Notably, this strain has multiple virulence factors including *iutA-iucABCD* (encoding aerobactin), *irp-ybtAEPQSUX* (encoding yersiniabactin), and mucoid regulators *rmpA* and *rmpA2* (Table 1). The virulence factor profile is in line with that commonly used to define hypervirulence (11).

**TABLE 1:** The antimicrobial resistance and virulence determinants of CRKP strain 141091

Gene	Product	Class
Antimicrobial resistance		
*rmtB1*	16S rRNA (guanine(1405)-N(7))-methyltransferase	Aminoglycoside
*bla*_LAP-2_	Class A β-lactamase	β-lactam
*bla*_SHV-11_	Broad-spectrum class A β-lactamase	β-lactam
*bla*_TEM-1_	Broad-spectrum class A β-lactamase	β-lactam
*bla*_KPC-2_	Carbapenem-hydrolyzing class A β-lactamase	Carbapenem
*bla*_CTX-M-65_	Extended-spectrum class A β-lactamase	Cephalosporin
*catA2*	Type A-2 chloramphenicol O-acetyltransferase	Chloramphenicol
*fosA*	FosA5 family fosfomycin resistance glutathione transferase	Fosfomycin
*qnrS1*	Quinolone resistance pentapeptide repeat protein	Quinolone
*aadA2*	ANT(3'')-Ia family aminoglycoside nucleotidyltransferase	Streptomycin
*sul2*	Sulfonamide-resistant dihydropteroate synthase	Sulfonamide
*tet(A*)	Tetracycline efflux MFS transporter	Tetracycline
*dfrA14*	Trimethoprim-resistant dihydrofolate reductase	Trimethoprim
Virulence (-allele no.)		
*iucA-1*	N(2)-citryl-N(6)-acetyl-N(6)-hydroxylysine synthase	Aerobactin
*iucB-1*	N(6)-hydroxylysine O-acetyltransferase	Aerobactin
*iucC-1*	Aerobactin synthase	Aerobactin
*iucD-1*	L-lysine N6-monooxygenase	Aerobactin
*iutA-1*	Ferric aerobactin receptor	Aerobactin
*iutA-41*	Ferric aerobactin receptor	Aerobactin
*rmpA-2*	Hypermucoidy marker gene	Hypermucoidy
*rmpA2-3*	Capsular polysaccharide synthesis activator	Hypermucoidy
*mrkA-2*	Fimbrial subunit type 3	Type 3 fimbriae
*mrkB-2*	Chaperone protein MrkB	Type 3 fimbriae
*mrkC-2*	Uter membrane usher protein MrkC	Type 3 fimbriae
*mrkD-12*	Fimbria adhesin protein	Type 3 fimbriae
*mrkF-8*	Fimbriae stability-related protein MrkF	Type 3 fimbriae
*mrkH-7*	Transcriptional regulatory protein	Type 3 fimbriae
*mrkI-15*	LuxR family regulatory protein	Type 3 fimbriae
*mrkJ-12*	Phosphodiesterase MrkJ	Type 3 fimbriae
*fyuA-11*	Pesticin/yersiniabactin receptor FyuA	Yersiniabactin
*irp1-65*	Yersiniabactin biosynthetic protein Irp1	Yersiniabactin
*irp2-305*	Yersiniabactin biosynthetic protein Irp2	Yersiniabactin
*ybtA-1*	Yersiniabactin transcriptional regulator YbtA	Yersiniabactin
*ybtE-5*	Yersiniabactin siderophore biosynthetic protein YbtE	Yersiniabactin
*ybtP-5*	Lipoprotein inner membrane ABC-transporter	Yersiniabactin
*ybtQ-6*	Putative ABC transporter protein	Yersiniabactin
*ybtS-140*	Salicylate synthetase	Yersiniabactin
*ybtT-5*	Yersiniabactin biosynthetic protein YbtT	Yersiniabactin
*ybtU-9*	Thiazolinyl-S-HMWP1 reductase YbtU	Yersiniabactin
*ybtX-11*	Putative cytoplasmic transmembrane protein YbtX	Yersiniabactin

In summary, we reported the genome sequence of an ST11-KL62 CRKP strain carrying multiple virulence determinants. To the best of our knowledge, ST11-KL62 *K. pneumoniae* has not been reported in the literature yet and may represent a new sublineage within ST11 in addition to KL47 and KL64. The origin of ST11-KL62 type warrants investigation.

## Data Availability

The genome assembly of strain 141091 has been deposited in GenBank under the accession number JBEFAU000000000. The raw sequence reads are available in the Sequence Read Archive (SRA) database under accession number SRR30336424.

## References

[1] Podschun R, Ullmann U. 1998. Klebsiella spp. as nosocomial pathogens: epidemiology, taxonomy, typing methods, and pathogenicity factors. Clin Microbiol Rev 11:589–603. doi:10.1128/CMR.11.4.589 9767057 PMC88898

[2] Wang G, Zhao G, Chao X, Xie L, Wang H. 2020. The characteristic of virulence, biofilm and antibiotic resistance of Klebsiella pneumoniae. Int J Environ Res Public Health 17:6278. doi:10.3390/ijerph17176278 32872324 PMC7503635

[3] Shi Q, Ruan Z, Zhang P, Hu H, Han X, Wang Z, Lou T, Quan J, Lan W, Weng R, Zhao D, Du X, Yu Y, Jiang Y. 2024. Epidemiology of carbapenem-resistant Klebsiella pneumoniae in China and the evolving trends of predominant clone ST11: a multicentre, genome-based study. J Antimicrob Chemother 79:2292–2297. doi:10.1093/jac/dkae227 38997220

[4] Hu F, Pan Y, Li H, Han R, Liu X, Ma R, Wu Y, Lun H, Qin X, Li J, Wang A, Zhou M, Liu B, Zhou Z, He P. 2024. Carbapenem-resistant Klebsiella pneumoniae capsular types, antibiotic resistance and virulence factors in China: a longitudinal, multi-centre study. Nat Microbiol 9:814–829. doi:10.1038/s41564-024-01612-1 38424289 PMC10914598

[5] Bolger AM, Lohse M, Usadel B. 2014. Trimmomatic: a flexible trimmer for Illumina sequence data. Bioinformatics 30:2114–2120. doi:10.1093/bioinformatics/btu170 24695404 PMC4103590

[6] Shen W, Le S, Li Y, Hu F. 2016. SeqKit: a cross-platform and ultrafast toolkit for FASTA/Q file manipulation. PLoS ONE 11:e0163962. doi:10.1371/journal.pone.0163962 27706213 PMC5051824

[7] Bankevich A, Nurk S, Antipov D, Gurevich AA, Dvorkin M, Kulikov AS, Lesin VM, Nikolenko SI, Pham S, Prjibelski AD, Pyshkin AV, Sirotkin AV, Vyahhi N, Tesler G, Alekseyev MA, Pevzner PA. 2012. SPAdes: a new genome assembly algorithm and its applications to single-cell sequencing. J Comput Biol 19:455–477. doi:10.1089/cmb.2012.0021 22506599 PMC3342519

[8] Jain C, Rodriguez-R LM, Phillippy AM, Konstantinidis KT, Aluru S. 2018. High throughput ANI analysis of 90K prokaryotic genomes reveals clear species boundaries. Nat Commun 9:5114. doi:10.1038/s41467-018-07641-9 30504855 PMC6269478

[9] Lam MMC, Wick RR, Watts SC, Cerdeira LT, Wyres KL, Holt KE. 2021. A genomic surveillance framework and genotyping tool for Klebsiella pneumoniae and its related species complex. Nat Commun 12:4188. doi:10.1038/s41467-021-24448-3 34234121 PMC8263825

[10] Feldgarden M, Brover V, Gonzalez-Escalona N, Frye JG, Haendiges J, Haft DH, Hoffmann M, Pettengill JB, Prasad AB, Tillman GE, Tyson GH, Klimke W. 2021. AMRFinderPlus and the reference gene catalog facilitate examination of the genomic links among antimicrobial resistance, stress response, and virulence. Sci Rep 11:12728. doi:10.1038/s41598-021-91456-0 34135355 PMC8208984

[11] Russo TA, Marr CM. 2019. Hypervirulent Klebsiella pneumoniae. Clin Microbiol Rev 32:e00001–e00019. doi:10.1128/CMR.00001-19 31092506 PMC6589860

